# Supplementary material for: Hydrocarbon Sorption in Flexible MOFs—Part II: Understanding Adsorption Kinetics
Source: Nanomaterials (Basel). 2023 Feb 2;13(3):601. doi: 10.3390/nano13030601 (PMC9919684; doi:10.3390/nano13030601)
Supplement: Supplementary file 1 [file nanomaterials-13-00601-s001.zip › nanomaterials-2193397-supplementary.pdf]

Supplementary Materials

Hydrocarbon Sorption in Flexible MOFs—Part II: Understanding Adsorption Kinetics

Hannes Preißler-Kurzhöfer [1,2,*], Andrei Kolesnikov [2], Marcus Lange [2], Jens Möllmer [2], Oliver Erhart [3], Merten Kobalz [3], Seungtaik Hwang [4], Christian Chmelik [4], Harald Krautscheid [3] and Roger Gläser [1,*]

[1] Institut für Technische Chemie, Fakultät für Chemie und Mineralogie, Universität Leipzig, Linnéstraße 3, D-04103 Leipzig, Germany
[2] Institut für Nichtklassische Chemie e.V., Universität Leipzig, Permoserstraße 15, D-04318 Leipzig, Germany
[3] Institut für Anorganische Chemie, Fakultät für Chemie und Mineralogie, Universität Leipzig, Johannisallee 21, D-04103 Leipzig, Germany
[4] Fakultät für Physik und Geowissenschaften, Universität Leipzig, Linnéstraße 5, D-04103 Leipzig, Germany
* Correspondence: che09bcz@studserv.uni-leipzig.de (H.P.-K.); roger.glaeser@uni-leipzig.de (R.G.)

S1. Isotherm fitting and gate-opening boundaries

Within this work, a dual-Dubinin-Asthakov equation (dual-DA) suitable for inhomogeneous adsorbents with two adsorption spaces was applied to model all experimental isotherms [1].

$$W = W_{0,1}\, e^{\left(\frac{-A}{E_1}\right)^{m_1}} + W_{0,2}\, e^{\left(\frac{-A}{E_2}\right)^{m_2}} \tag{S1}$$

Herein, W_0 is the saturation loading, while E and m mark the specific adsorption energy and the inhomogeneity parameter respectively. Since it is a dual-volume model, all three parameters are denoted with either 1 or 2. The fits for the *n*-butane adsorption on **1** are presented in Figure S1, the fitting parameters are in Table S1.

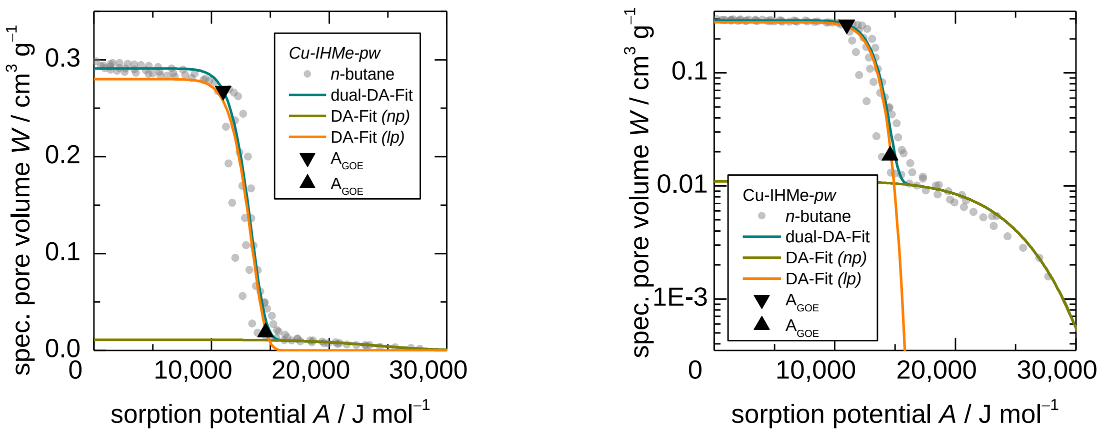

Figure S1: Fitting approach using the dual-DA equation for *n*-butane on **1** and the resulting gate-opening boundaries; left: linear scale, right: logarithmic scale.

Table S1: Fitting parameters for the dual-Dubinin-Asthakov model (dual-DA-Fit) for the ad- and desorption of *n*-butane on **1**.

Process	Pore phase *i*	$W_{0,i}$ / cm³ g⁻¹	E_i / J mol⁻¹	n_i
ADS	1 (narrow pore phase *np*)	0.011	-25000	6
ADS	2 (large pore phase mp)	0.28	-13500	12
DES	1 (narrow pore phase *np*)	0.011	-25000	6
DES	2 (large pore phase mp)	0.28	-17500	20

After fitting, essential points within the isotherms can be specified. For flexible MOFs, it is common that one gate-opening pressure is defined. However, due to particle size distributions of the adsorbents, different energy barriers for the structural transition can occur and therefore, pressure ranges rather than isobaric phase changes are observed. Thus, in this work, two pressure points are defined to describe the entire gate-opening. For flexible MOFs, the first term in the dual-DA Equation (S1) describes the sorption in the narrow

pore phase (*np*-phase), while the second term describes the structural transition and medium pore phase (*mp*-phase) sorption. The gate-opening starts when the latter term starts adding significant pore volume. Therefore, to find a gate-opening start (GOS) point in the isotherm, a boundary value of 0.01 $cm^3 g^{-1}$ for the *mp*-phase term was set.

Secondly, a gate-opening end (GOE) is determined, herein defined using the excess surface work (ESW). The ESW, usually computed as the product of the adsorbed amount and the change in chemical potential, gives rise to the strength of interaction between the sorbent and the sorptive according to Adolphs [2]. It is mathematically identical to the first derivative of the surface potential as defined by Myers and Prausnitz [3]. It describes the counteracting processes of decreasing surface free energy and increasing isothermal and isobaric work of sorption. The occurring minimum in the plots can then be identified as the completion of a monolayer.
In this work, the product of the adsorption potential and the volumetric loading was used to identify the position of a quasi-monolayer at an ESW-maximum (see Figure S2). It has to be stated that the resulting ESW-values are not of relevance in this case but just the position of the extrema. The resulting values for the GOS and GOE points are summarized in Table S2.

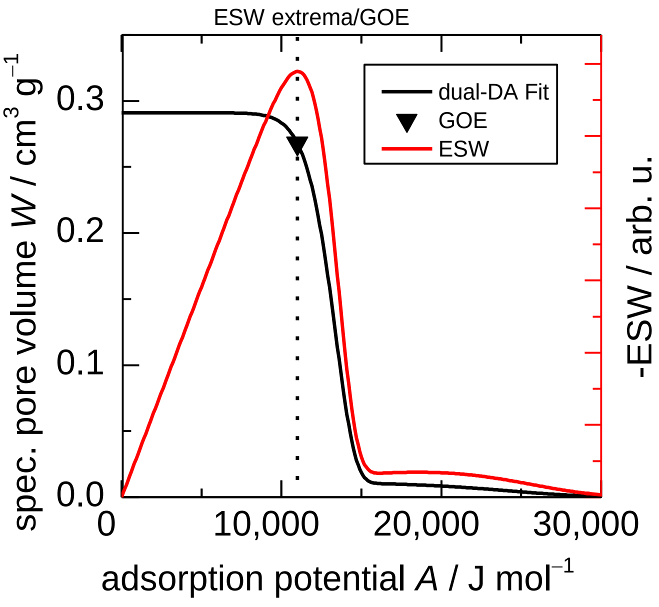

Figure S2: Determination of gate-opening end (*GOE*) using the excess surface work (ESW) theory.

Table S2: Overview of the adsorption potential, the corresponding loading and pressures for the gate-opening start (*GOS*) and gate-opening end (*GOE*) boundaries.

	gate-opening start (GOS)	gate-opening end (GOE)
Adsorption potential A / J mol^{-1}	14600	10600
Loading W / $cm^3 g^{-1}$	0.019	0.268

p (283 K) / kPa	0.3	1.3
p (298 K) / kPa	0.7	2.9
p (313 K) / kPa	1.3	5.5
p (328 K) / kPa	3.0	10.0

Excursion Desorption:

In the same way as shown for the adsorption, the desorption can be fitted as well. As stated in the main manuscript, the desorption of the system under study appears to be closer to the real thermodynamic equilibrium. This is underlined by (a) the much narrower desorption range (given the isotherm at 298 K, the gate-opening refers to pressures between 6 and 25 mbar while the gate-closing to 7 and 1 mbar) and (b) by the higher transport diffusivities that seemingly are not influenced as much during the gate-closing as compared to the gate-opening. For a more detailed assessment, the reader is referred to Preißler-Kurzhöfer et al. [4].

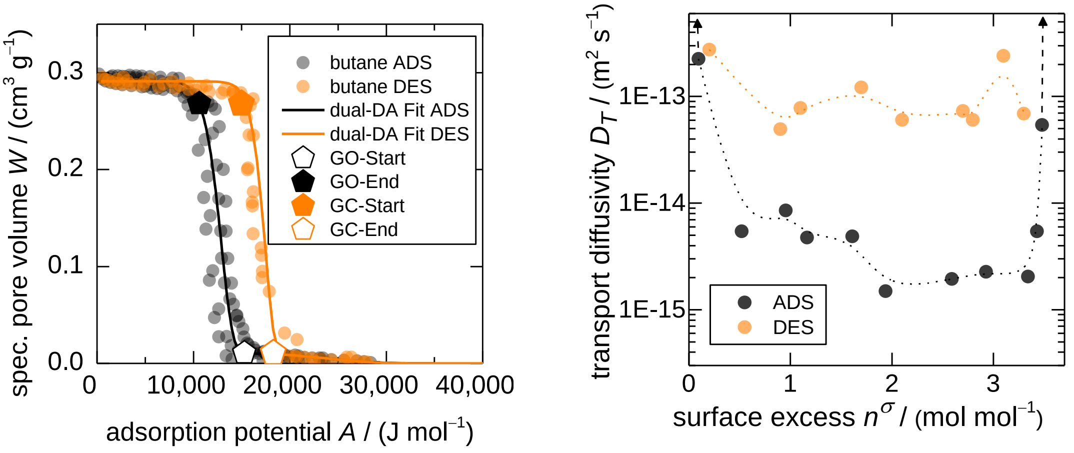

Figure S3: Characteristic patterns for ad- and desorption for n-butane on **1** with their respective DA-fits and structural transition boundaries (**left**). Furthermore, the overall transport diffusivity in dependence of the molar loading is displayed as measured by IR microscopy (**right**).

S2. Deduction of sorption characteristics

The *n*-butane uptakes on **1** for a pressure increases of 0→10 kPa, 0→15 kPa and 0→30 kPa at 298 K are herein further analysed to deduct information about the kinetics of the system (Figure S3). Firstly, plots of ln(1-Ψ) (Ψ: relative uptake) as a function of time give rise to the existence of possible non-isothermal behaviour during the uptake [5]. Figure S4 (right) shows a smooth curvature for the 0→10 kPa uptake, which is unusual for adsorption processes and is herein explained with the dependence of the rate of structural transition on the particle size distribution. This argument is further underlined in Figure S5. In a ln(1-Ψ)-plot, the states of structural transition in dependence of time, taken from the *in situ* XRD measurements show a qualitatively similar long-term behaviour as the gravimetric uptake, leading to the conclusion that the gate-opening itself is the rate-determining step in the whole process.

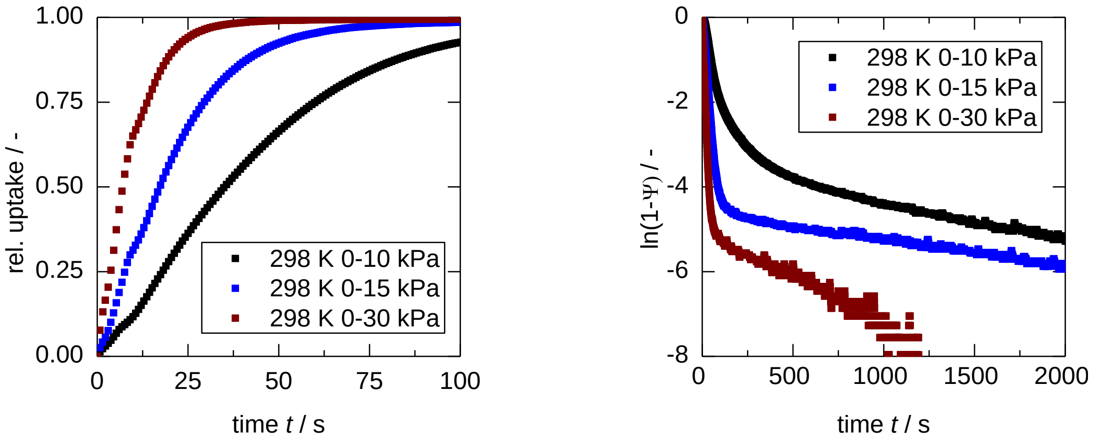

Figure S4: Uptake curves of *n*-butane on **1** at 298 K using different pressure steps in the normal representation (**left**) and in a ln(1-Ψ) representation (**right**).

The uptakes at 0→15 kPa and 0→30 kPa, however, show very strong kinks in their shape, which typically indicates non-isothermal behaviour. From a side experiment, where the temperature of a 1 cm^3 bed of **1** was monitored during the uptake of *n*-butane, it can be seen that the temperature does indeed increase during the process, also for small pressure steps (Figure S6). However, temperature changes of around 2–4 K would not decrease the overall sorptive loading significantly given the adsorption isotherms. This is further underlined by the fact that a value of ln(1-ψ)=–5, the point at which the kink occurs and the non-isothermal behaviour becomes evident, corresponds to a relative uptake of ~0.993. Thus, all uptake curves in this work can satisfyingly be modelled with the isothermal equation presented here. For larger adsorption columns, this may be different and non-isothermal models would be necessary. For the case of the uptake at 283 K with a pressure step 0→60 kPa, a possible non-isothermal model is described in the next section.

Additionally, in Figure S7, the uptake at 298 K and a pressure step of 0→10 kPa, represented as relative uptake as a function of the square root of time, displays a linear range followed by an inflection point. This is a more emphasized view on the inflection point caused by the surface diffusion on the particles, as stated in the main manuscript.

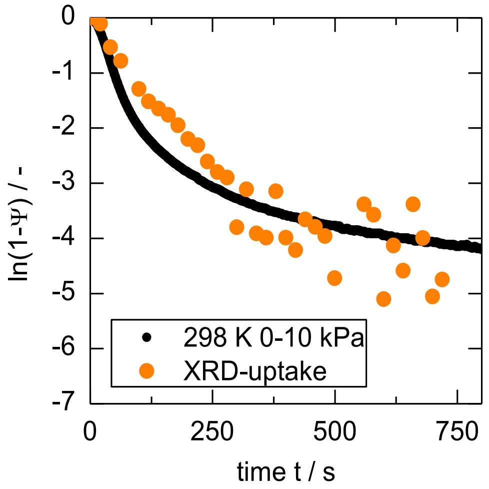

Figure S5: Gravimetric and XRD-uptake curves for 298 k and 0→10 kPa on **1** in a ln(1-**Ψ**) representation.

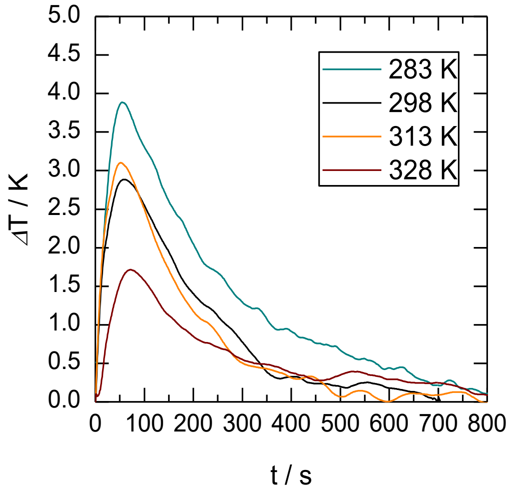

Figure S6: Temperature curves during the adsorption of 0.04 p p_0^{-1} of n-butane at 283, 298, 313 and 328 K on a 1 cm^3 bed of **1**.

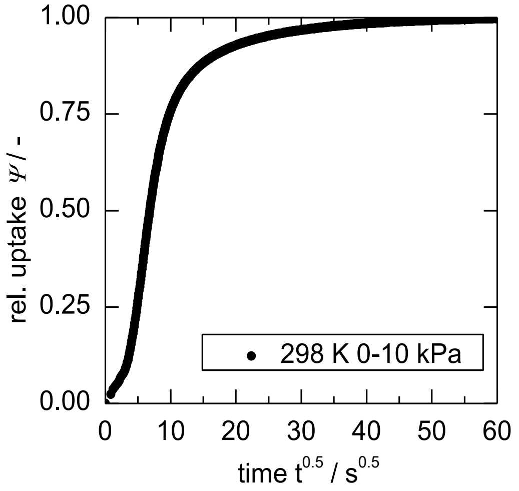

Figure S7: Uptake curve of *n*-butane on **1** at 298 K with a pressure step 0→10 kPa as a function of the square root of time.

Uptake fitting for larger pressure Jumps

As reported in the manuscript, pressure steps that largely overdose the GOE attain more regular shapes. All three rate constants of the extended Tanaka-model for the second set of uptake experiments shown in the main manuscript are listed in Table S3. As becomes apparent, k_{GO} is increasing with higher pressure and higher temperature and becomes less of a limiting factor for the overall velocity of gas uptake. Furthermore, the uptakes can then even be partially modelled with a simple LDF-approach (Figure S8), the relevant singular k_{LDF}-parameters are tabulated in the last column in Table S3.

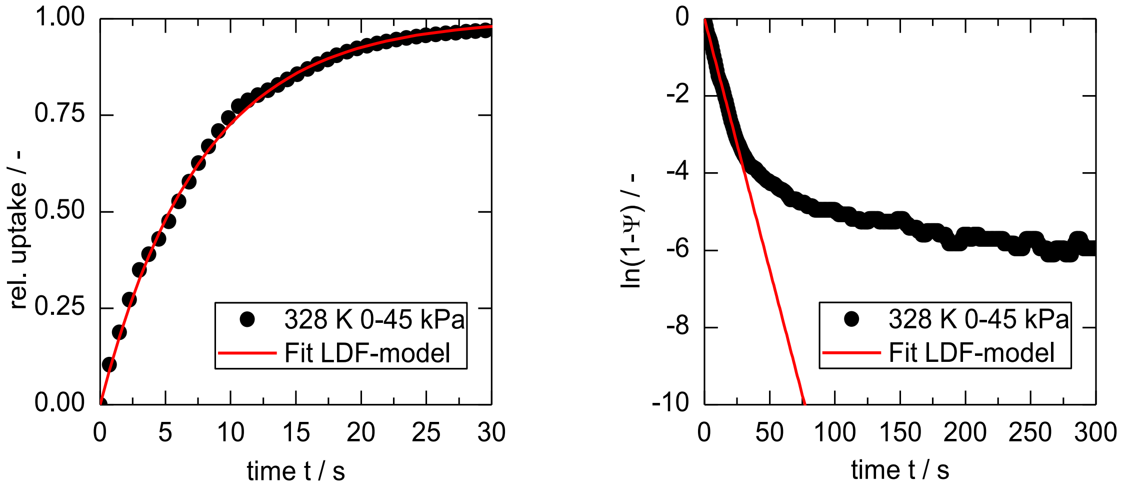

Figure S8: Uptake at 328 K and 0→45 kPa fitted with a simple LDF-model in conventional visualization (relative uptake vs. time) (**left**) and logarithmic form of the residual uptake for insights into the long-term behavior (**right**) .

From these values, it is possible to examine conditions for the temperature dependency crossover, at which Arrhenius behaviour switches to anti Arrhenius behaviour due to the relative position of the gas uptake pressure to the GOE. Therefore, all k_{LDF} values of the second set of experiments were plotted in a *ln k* vs 1/T-plot and the iso-chemical potential lines visualized (see Figure S9). Exemplary, an arbitrary value of ln k_{LDF} = −2.75 is picked in the so-called Arrhenius-plot and the experimental conditions lying on the *iso*-chemical potential lines are calculated. Both, temperature and pressure are higher on the dA-line of 4100 J mol^{-1}, although the overall velocity of adsorption is the same.

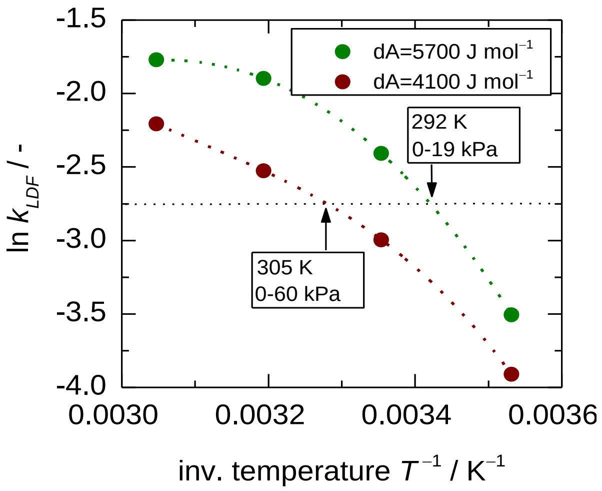

Figure S9: Arrhenius plot for the k_{LDF} fits on the gas uptakes at the iso-chemical potential lines dA=4100 J mol^{-1} and dA=5700 J mol^{-1} and the experimental conditions for a crossover in the temperature dependency of the overall velocity of adsorption.

Table S3: Overview over all relevant fitting parameters

dA=4100 J mol^{-1}	k_{D1}	k_{D2}	k_{GO}	k_{LDF}
283 K / 0→10 kPa	0.05	0.05	0.03	0.02*
298 K / 0→18 kPa	0.30	0.11	0.06	0.04
313 K / 0→30 kPa	0.40	0.10	0.13	0.06
328 K / 0→50 kPa	0.70	0.20	0.28	0.11

dA=5700 J mol^{-1}	k_{D1}	k_{D2}	k_{GO}	k_{LDF}
283 K / 0→18 kPa	0.60	0.08	0.05	0.03
298 K / 0→35 kPa	0.70	0.19	0.16	0.09
313 K / 0→60 kPa	1.00	0.30	0.22	0.15
328 K / 0→100 kPa	1.00	0.30	0.22	0.17

* LDF-model does not fit this uptake well in the lower region, only long-term behaviour was fitted

S3 Additional Structural Data and Figures

Table S4: Crystal structure data and Rietveld refinement parameters of **1** for the *lp*-, *mp*- and *np*-form

	$[Cu_2(H\text{-}Me\text{-}trz\text{-}Ia)_2]$ (*lp*-form)	$[Cu_2(H\text{-}Me\text{-}trz\text{-}Ia)_2]$ (*mp*-form)	$[Cu_2(H\text{-}Me\text{-}trz\text{-}Ia)_2]$ (*np*-form)
	single crystal data [8]	crystal powder data	crystal powder data
Crystal system	monoclinic	monoclinic	monoclinic
Space group	*P* $2_1/c$ (no. 14)	*P* $2_1/c$ (no. 14)	*P* $2_1/c$ (no. 14)
Unit cell parameters / pm, °	a = 1085.9(1)<br>b = 1278.6(2)<br>c = 1402.6(2)<br>β = 110.508(8)	a = 1098.10(5)*<br>b = 1498.83(5)<br>c = 1174.66(5)<br>β = 120.138(3)	a = 1077.90(5)**<br>b = 1522.16(6)<br>c = 775.14(4)<br>β = 115.420(5)
Volume / 10^6 pm^3	1823.9(4)	1672.0(1)	1148.7(1)
Z	2	2	2
Density / g cm^{-3} (frame-work without guest molecules)	1.124	1.458	1.785
Calc. pore fraction / %	50	43	4
Pore Volume / cm^3 g^{-1}	0.451	0.294	0.023
R_{int}	0.0585		
R_1	0.0587		
wR_2	0.1597		
R_p		0.0668	0.0607
R_{wp}		0.0858	0.0750

* Unit cell parameters for conventional setting: *a* = 1098.10(5), *b* = 1498.83(5), *c* = 1135.94(5), *β* = 116.582(3), space group *P*$2_1/c$

** Unit cell parameters for conventional setting: *a* = 1022.49(5), *b* = 1522.16(6), *c* = 775.14(4) pm, *ß* = 107.792(5)°

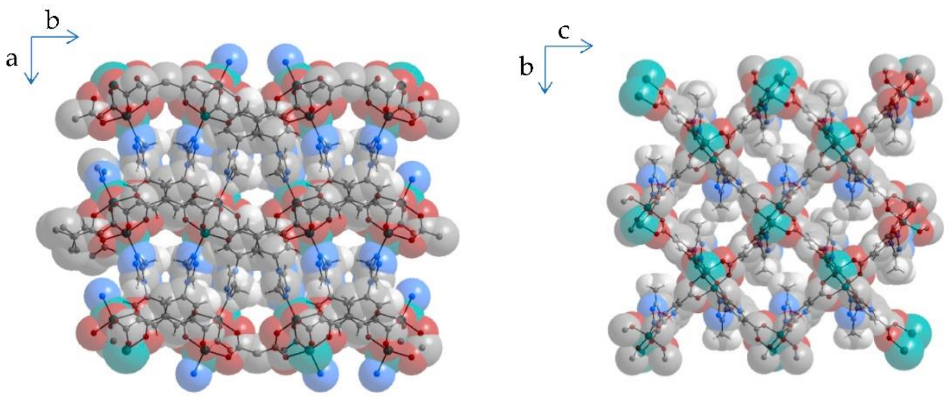

Figure S10: Parallel projection of Cu-IHMe-pw along the largest cavities in the np form (**left**) and the mp form (**right**), atoms shown with van der Waals radii (taken from Preißler-Kurzhöfer et al. [4]).

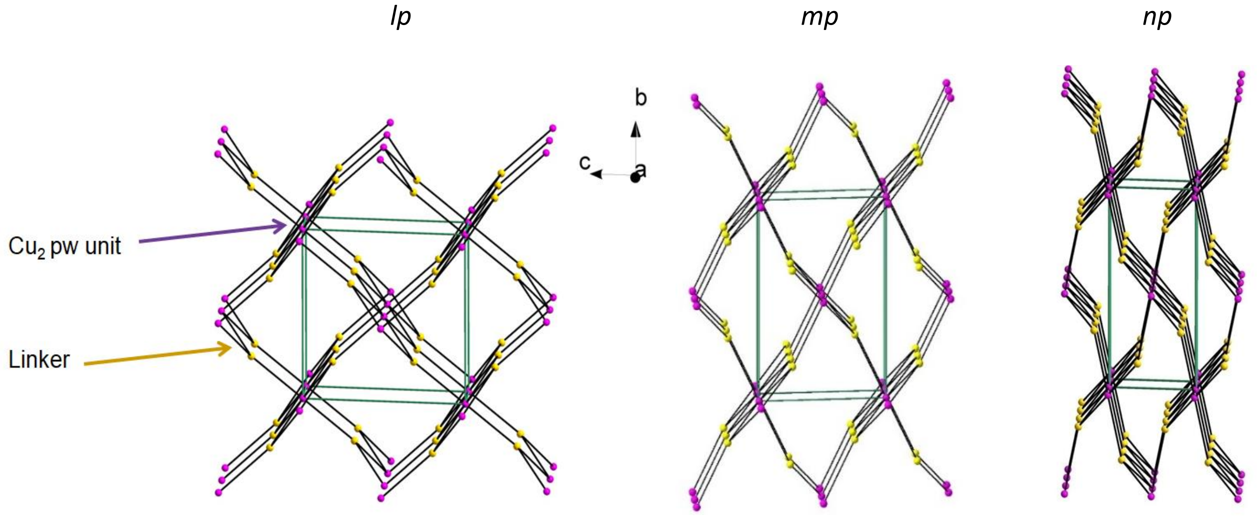

Figure S11: Unit cell diagrams of the simplified crystal structure of the *lp*-, *mp*- and *np*-phase of [Cu_2(H-Me-trz-Ia)$_2$] (1). The violet spheres symbolize the centres of the Cu_2 paddle wheel units, the yellow spheres represent the centers of the H-Me-trz-ia^{2-} linkers.

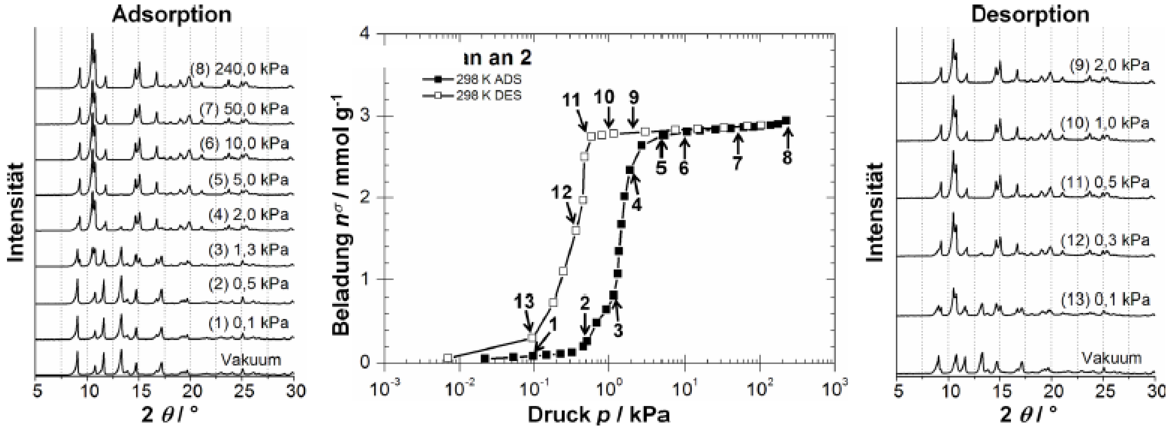

Figure S12: Adsorption isotherm for *n*-butane on **1** at 298 K (**center**) and the corresponding diffraction patterns for adsorption (**left**) and desorption (**right**).

S4. Further Figures

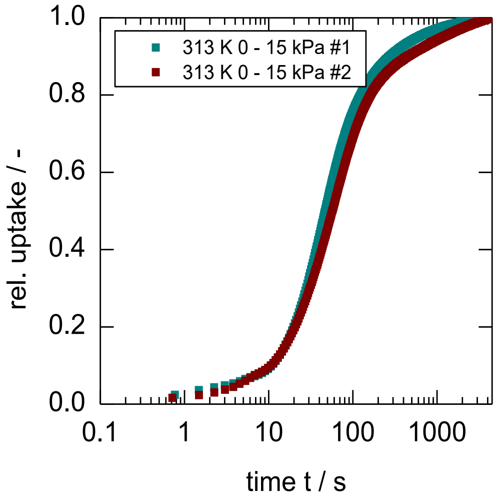

Figure S13: Reproducibility of the *n*-butane uptake on **1** from 0→15 kPa at 313 K via gravimetry.

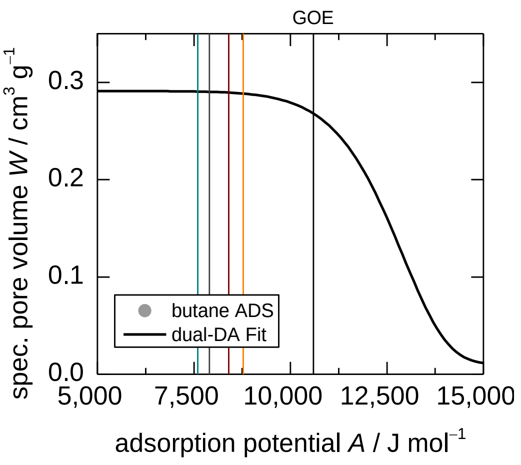

Figure S14: Relative positions of gas uptakes 0→0.04 relative pressure in regard to the *GOE* for 283 K (turquoise), 298 K (black), 313 K (orange) and 328 K (red).

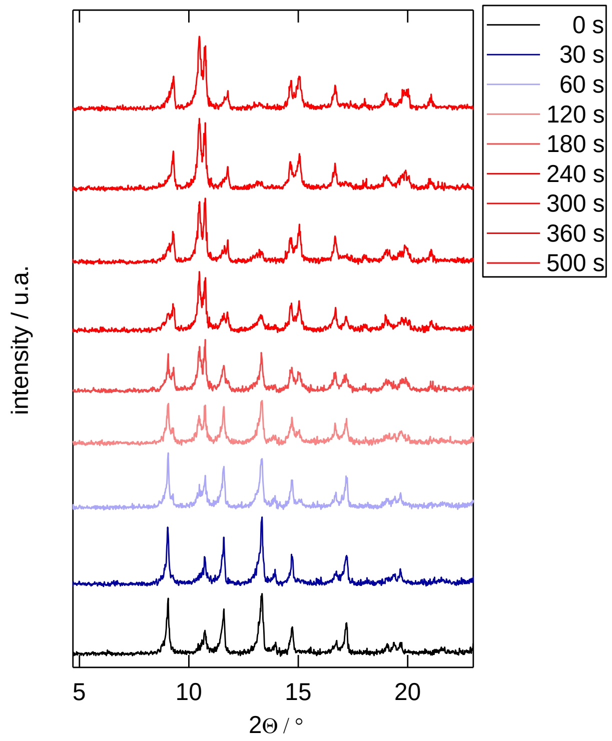

Figure S15: Time dependent X-ray powder diffraction patterns for the uptake of *n*-butane on **1** on the first run. Herein, the structural transition needed several minutes, most likely due to larger particle sizes.

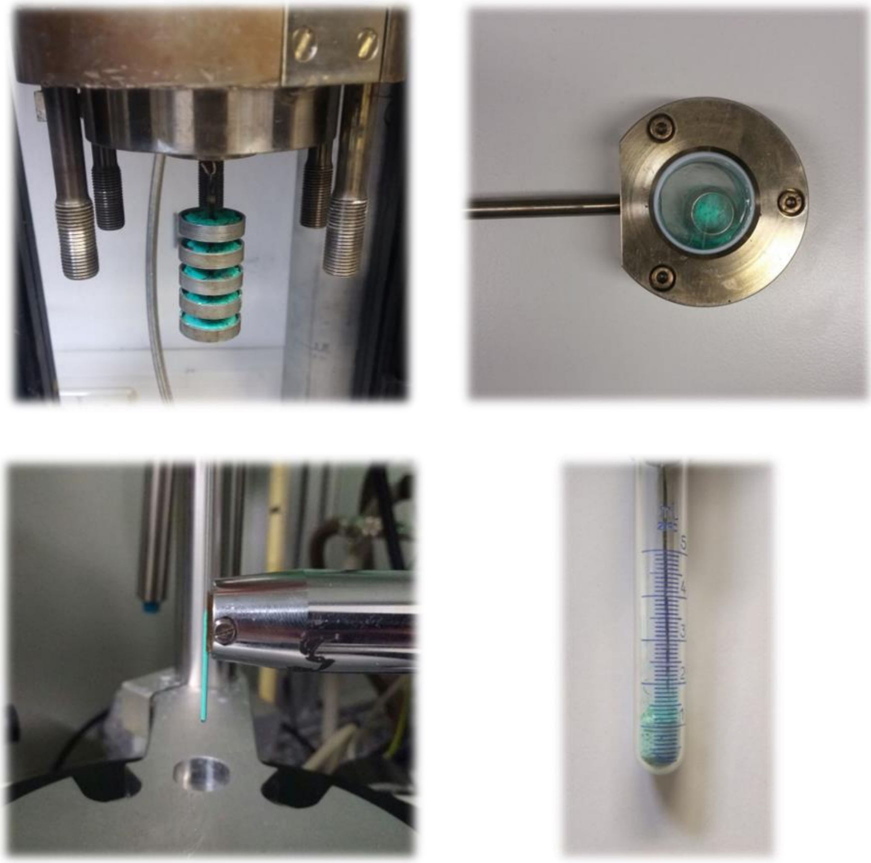

Figure S16: Images of the used set-ups for gravimetry (**upper left**), IR measurements (**upper right**), XRD (**lower left**) and bed-temperature measurements (**lower right**).

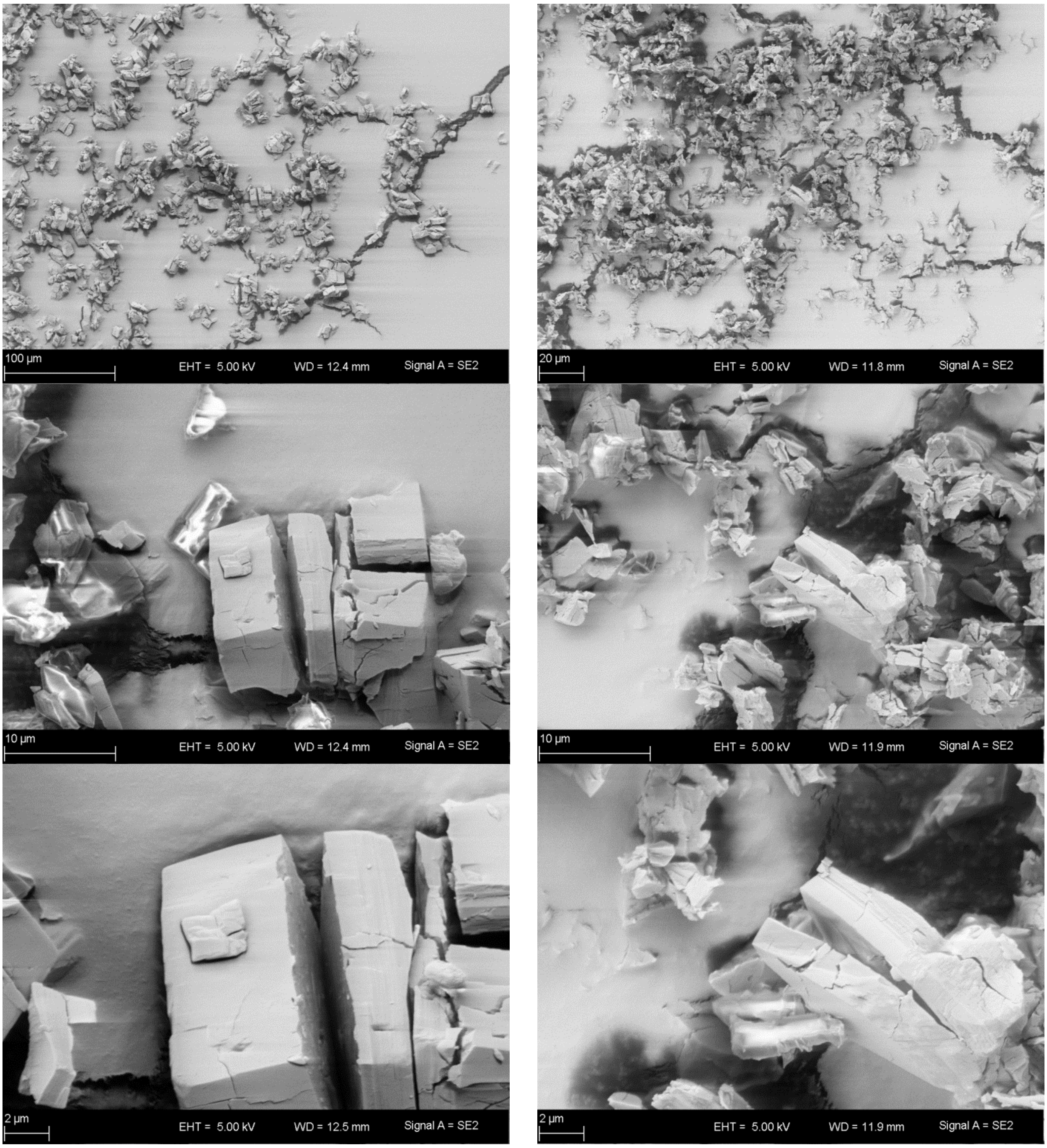

Figure S17: SEM images of a freshly synthesized, evacuated sample of **1** (**left**) and a sample of **1** after 20 adsorption-desorption cycles (**right**), showing more fractions and smaller particle sizes for the used material.

S5. References Supplementary Information

[1] Burevski, D. The application of the Dubinin-Astakhov equation to the characterization of microporous carbons. Colloid & Polymer Sci 1982, 260, 623–627, doi:10.1007/BF01422595.

[2] Adolphs, J. Excess surface work?: A modelless way of getting surface energies and specific surface areas directly from sorption isotherms. App. Surf. Sci. 2007, 253, 5645–5649, doi:10.1016/j.apsusc.2006.12.089.

[3] Myers, A.L. Thermodynamics of adsorption in porous materials. AIChE J. 2002, 48, 145–160, doi:10.1002/aic.690480115.

[4] Preißler-Kurzhöfer, H.; Lange, M.; Kolesnikov, A.; Möllmer, J.; Erhart, O.; Kobalz, M.; Krautscheid, H.; Gläser, R. Hydrocarbon Sorption in Flexible MOFs-Part I: Thermodynamic Analysis with the Dubinin-Based Universal Adsorption Theory (D-UAT). Nanomaterials 2022, 12, doi:10.3390/nano12142415.

[5] Möller, A.; Guderian, J.; Möllmer, J.; Lange, M.; Hofmann, J.; Gläser, R. Kinetische Untersuchungen zur Aufkonzentrierung methanhaltiger Gasgemische an Kohlenstoffmolekularsieben. CIT 2014, 86, 41–46, doi:10.1002/cite.201300067.

[6] Tanaka, D.; Nakagawa, K.; Higuchi, M.; Horike, S.; Kubota, Y.; Kobayashi, T.C.; Takata, M.; Kitagawa, S. Kinetic gate-opening process in a flexible porous coordination polymer. Angew. Chem. Int. Ed. 2008, 47, 3914–3918, doi:10.1002/anie.200705822.

[7] S. Sircar. Linear-driving-force Model for Non-isothermal Gas Adsorption Kinetics. J. Chem. Soc., Faraday Trans. I 1983, 785–796.

[8] Kobalz, M.; Lincke, J.; Kobalz, K.; Erhart, O.; Bergmann, J.; Lässig, D.; Lange, M.; Möllmer, J.; Gläser, R.; Staudt, R.; et al. Paddle wheel based triazolyl isophthalate MOFs: Impact of linker modification on crystal structure and gas sorption properties. Inorg. Chem. 2016, 55, 3030–3039, doi:10.1021/acs.inorgchem.5b02921.
